# Complete pathologic clearance with vismodegib in advanced basal cell carcinoma of the scalp with cranial invasion

**DOI:** 10.1016/j.jpra.2022.09.001

**Published:** 2022-09-09

**Authors:** Joseph Dodson, Ryan Kelm, Kevin Cavanaugh

**Affiliations:** a Rush University Medical Center, Chicago, IL, United States; b Rush University Medical Center, Department of Dermatology, Chicago, IL, United States

**Keywords:** Neoadjuvant, Advanced basal cell carcinoma, Cranium, Immunocompromised, Vismodegib, Hedgehog Pathway Inhibitor

## Abstract

The management of advanced basal cell carcinoma (BCC) can be challenging and often involves a multi-disciplinary approach with dermatologists, plastic surgeons, and oncologists. Standard therapy for advanced BCCs has historically involved prompt excision and radiation; however, in recent years, management strategies utilizing hedgehog pathway inhibitors as neoadjuvant therapy have gained popularity. While controversy regarding management recommendations still exists, we present a case of advanced BCC with cranial involvement in an immunocompromised patient where the use of neoadjuvant vismodegib led to a favorable outcome and, surprisingly, complete the pathologic clearance of the tumor.

## Introduction

Basal cell carcinoma (BCC) is the most common skin cancer, occurring in older populations with an age-adjusted prevalence of 343 per 100,000 people.^1^^,^^2^ Risk factors include cumulative ultraviolet light exposure, previous radiation therapy, immunosuppressive medications, and arsenic toxicity.^1^ Although these skin cancers are rarely associated with metastasis and mortality, local invasion and destruction can cause significant morbidity in a minority of patients.^1^

BCC is typically classified into two major categories: indolent growth subtypes and aggressive growth subtypes.^1^ Histopathologic features determine the classification, and while indolent subtypes typically present as small, non-invasive lesions, aggressive subtypes are associated with more substantial local invasion, increased likelihood of metastasis, and higher rates of recurrence.^1^ Factors, such as tumor classification, location, size, gross appearance, and immunocompromised status, determine the degree of treatment necessary.^3^

The primary treatment of non-invasive BCC is surgical excision or local destruction; however, in cases with a larger tumor burden or more advanced disease, other treatment options, including radiation therapy or targeted pharmacologic therapy may be necessary.^3^^,^^4^ Vismodegib is a hedgehog pathway inhibitor that was approved in 2012 based on evidence from the ERIVANCE trial. In this trial, 104 patients with locally advanced BCC were treated with vismodegib 150 mg oral daily, and objective response rates were seen in 48% of patients.^5^ With this degree of success, vismodegib has provided an alternative avenue for the treatment of advanced BCC, with the intent to downstage tumors prior to surgical excision, termed neoadjuvant vismodegib. We present a case of an immunocompromised patient with advanced BCC involving a special site treated with vismodegib with complete pathologic response.

## Case

A 53-year-old female with Crohn's disease and Hurley stage 3 hidradenitis suppurativa (HS) presented with a large, ill-defined, fungating mass on the scalp greater than 11 cm in diameter, which had been present for approximately one year. She presented to our dermatology clinic for the management of poorly controlled HS; it was unclear why this lesion had not been biopsied and treated. Her immune suppression at that time included oral azathioprine 200 mg daily for well-controlled Crohn's disease, and Humira 40 mg subcutaneous (SC) weekly for HS. She stated that the scalp lesion had been growing rapidly, had become malodorous, and was very painful. She denied unintentional weight loss, fevers, chills, or shortness of breath.

**Figure 1:**
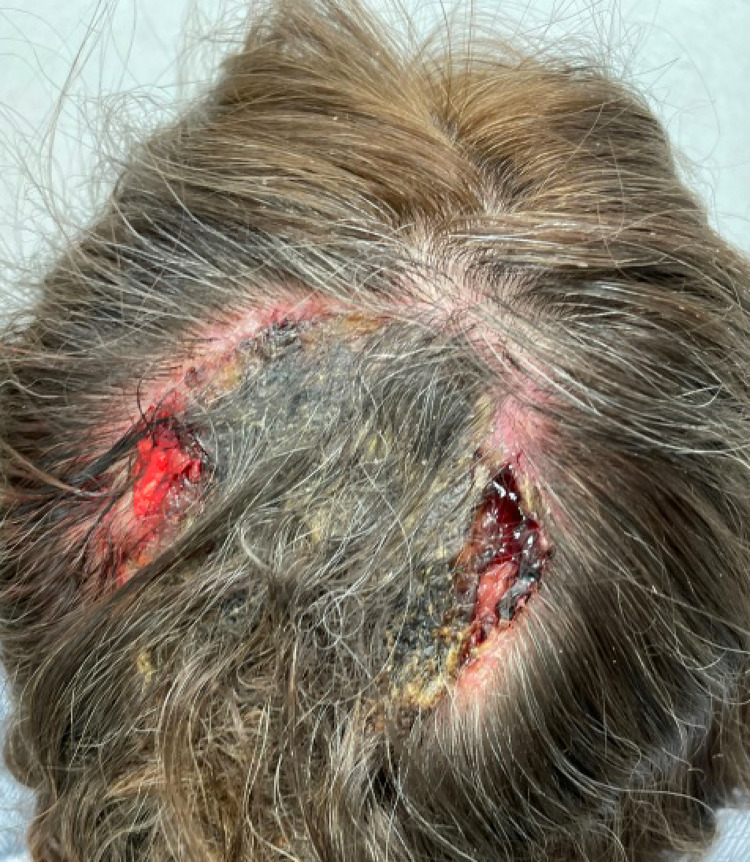
BCC of the scalp prior to initiation of vismodegib.

**Figure 2:**
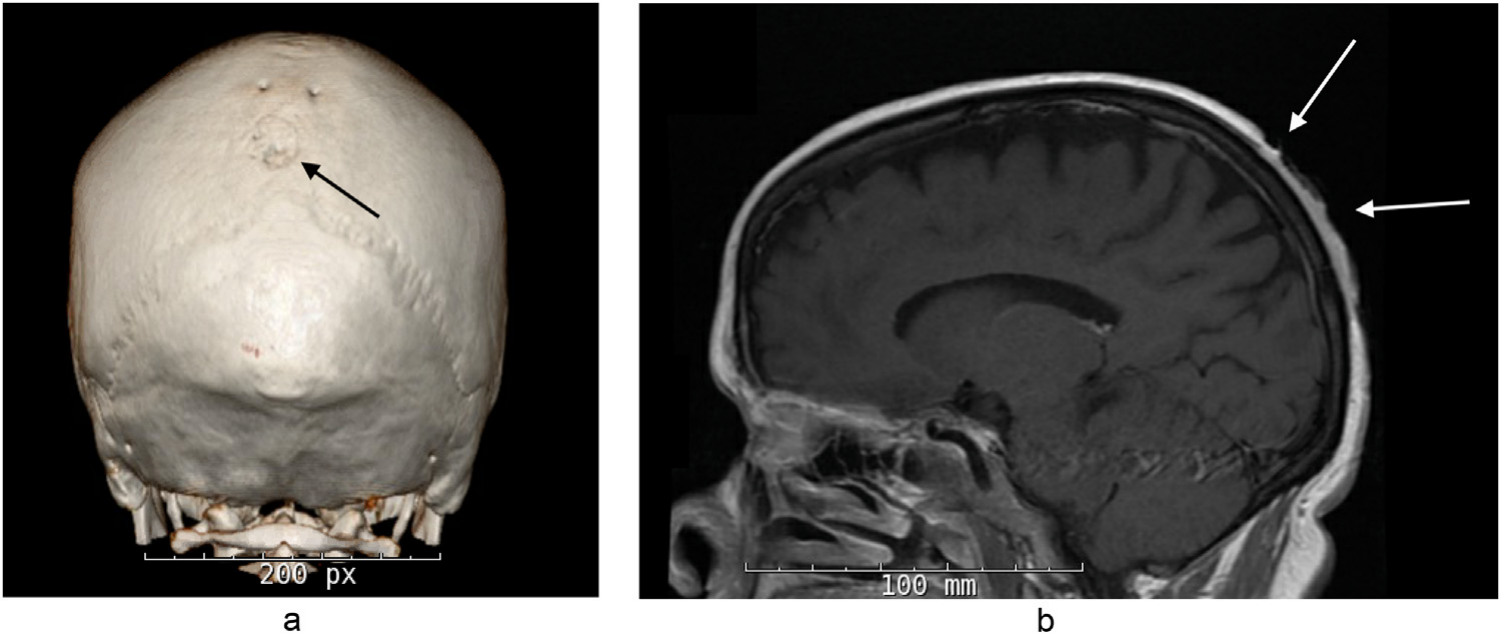
a: CT scan converted to 3D model depicting bone erosions of the cranium. b: MRI demonstrating cranial invasion.

Exam revealed that an 11.0 × 6.0 cm eroded heme-crusted mass encompassing the bulk of the vertex scalp and extending posteriorly to the occipital scalp [INSERT Figure 1]**.** A biopsy was performed and confirmed BCC nodular type. Further workup included CT and MRI, both of which demonstrated cranial invasion [INSERT Figure 2a and 2b]. She was discussed at our multi-disciplinary tumor board. Treatment options included immediate tumor resection with adjuvant radiation therapy (XRT) versus a trial of neoadjuvant vismodegib 150 mg daily prior to surgical resection and XRT. Ultimately, a decision was made to initiate vismodegib 150 mg daily with monthly follow-up for a period of three to four months to track clinical response. Azathioprine and Humira were discontinued. Given uncontrolled HS and co-morbid Crohn's disease, she was started on Stelara 90 mg SC every eight weeks concurrently with vismodegib**.**

**Figure 3:**
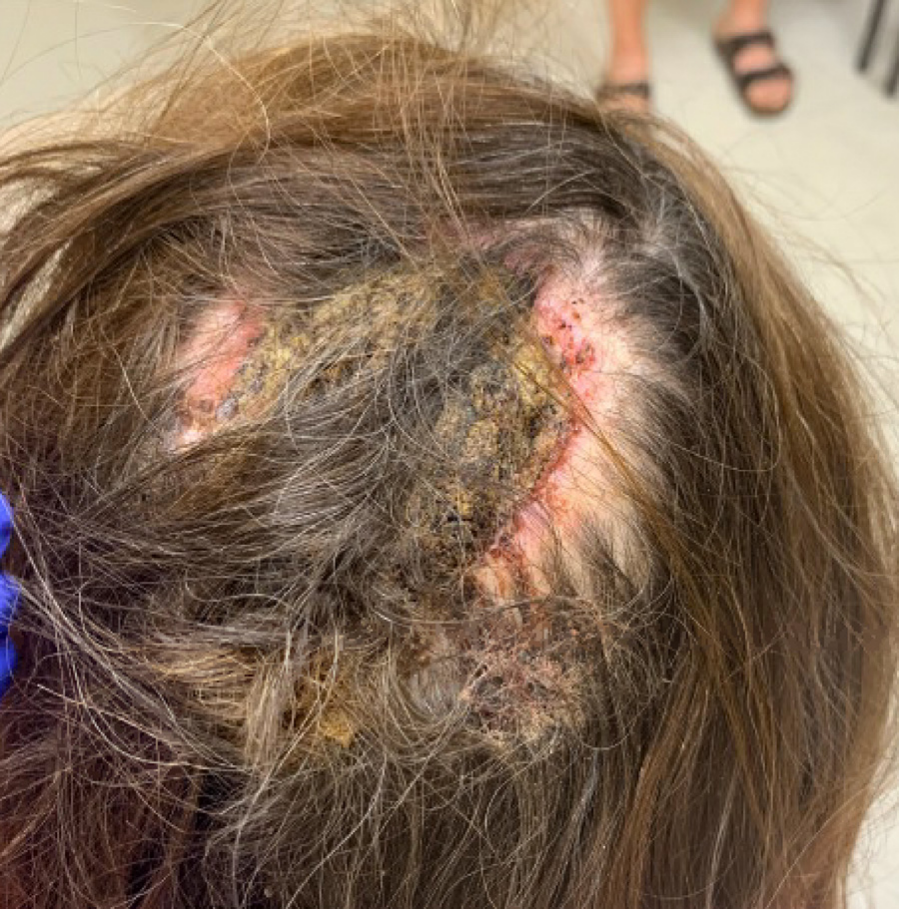
BCC of the scalp after 12 weeks of treatment with vismodegib.

**Figure 4:**
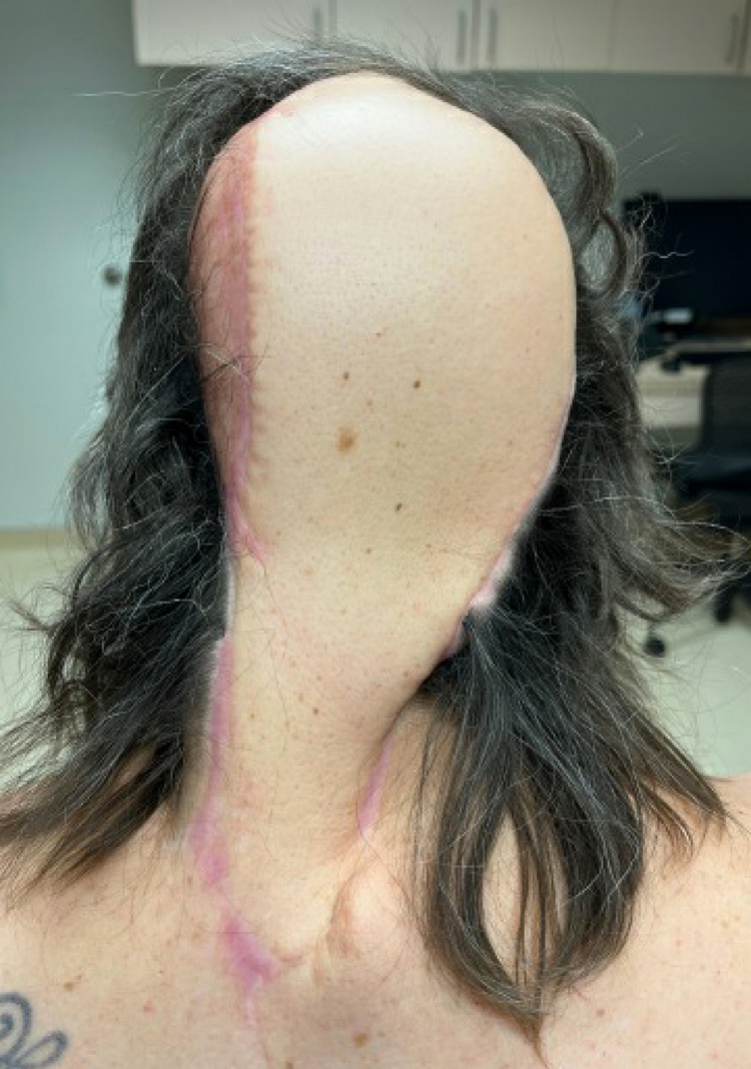
Clinical photograph of pedicled trapezius flap 9 months after surgery.

Upon the initiation of vismodegib, the lesion measured 11.0 × 6.0 cm. At 4, 8, and 12 weeks, the lesion measured 10.0 × 6.5 cm, 9.0 × 6.5 cm, and 8.8 × 6.3 cm, respectively [INSERT Figure 3]. At each month, the patient-reported improvement in gross appearance and pain symptoms. After 14 weeks of neoadjuvant vismodegib, the remaining lesion and involved cranium were resected with a 1 cm margin by surgical oncology, plastic surgery, and neurosurgery. The total size of the defect measured 8.5 × 11 cm with a cranial defect of 6 × 7 cm. Reconstruction involved a titanium mesh cranioplasty and pedicled trapezius flap [INSERT Figure 4]. Eight intraoperative frozen section biopsies to elucidate the margins of the tumor showed no evidence of BCC. Final pathology of the tissue and skull confirmed complete response to oral vismodegib. The clinically apparent mass measured prior to surgery was presumed to be necrotic tissue.

## Discussion

In most cases of BCC, surgical excision alone is the standard of care and performed with a high degree of success.^3^ However, in patients with advanced disease and/or special sites, standard treatment has not been fully defined.^3^^,^^4^ Radiation has been used to downstage tumors prior to surgery, but studies show suboptimal disease control.^3^^,^^4^ Neoadjuvant use of vismodegib has demonstrated clinical efficacy in advanced BCC, providing an 80% success rate in downstaging tumors prior to surgery.^6^ While the ERIVANCE trial showed that patients stayed on the medication for an average of 12.9 months without significant toxicity,^6^ more mild side effects often limit the medication's utility. Most patients will experience at least one adverse effect, including muscle spasms, fatigue, alopecia, and dyspepsia, leading to interruption of treatment in up to half of patients.^6^ Our patient experienced only mild side effects. Additionally, our patient's case displays the possibility of complete response in advanced BCC involving special sites with bony invasion. Interestingly, clinical response in such large tumors may be difficult to assess, as in our case, due to necrotic tissue presumed to be persistent BCC. For this reason, the lesion was not re-biopsied or re-staged. One could argue that this information could have resulted in a less extensive surgery. However, the consensus remained that, regardless of the clinical response, surgery was necessary to remove necrotic debris and sample the deep margins within the skull base.

Management of advanced BCC can be challenging and ideally involves a multi-disciplinary approach, including dermatologists, plastic surgeons, and oncologists, for best treatment results.^3^^,^^4^ In our case, consensus multi-disciplinary agreement on the use of neoadjuvant 150 mg daily vismodegib resulted in complete pathologic response and likely reduced the risk of potential surgical complications. While no trials have directly compared the response and recurrence rates of BCCs treated with and without adjunctive HHIT, the high response and low recurrence rates seen with adjunctive HHIT are considered superior to those achieved with traditional therapies alone.^7^ Additionally, most recurrences are seen after discontinuation of HHIT, demonstrating that vismodegib monotherapy without surgery is not recommended.^8^ Optimal management strategies continue to be discussed, designed, and tested for advanced BCC. Pharmacologic treatment with vismodegib or other hedgehog pathway inhibitors should be included in these conversations to best treat these patients.

## Funding sources

No funding sources were secured for this study.

## Ethical approval

Not required.

## Patient consent

Verbal and written consent for the publication of this case along with the photographs provided was obtained.

## Declaration of Competing Interest

All authors report no conflict of interest.
